# Insomnia Phenotypes Based on Objective Sleep Duration in Adolescents: Depression Risk and Differential Behavioral Profiles

**DOI:** 10.3390/brainsci6040059

**Published:** 2016-12-13

**Authors:** Julio Fernandez-Mendoza, Susan L. Calhoun, Alexandros N. Vgontzas, Yun Li, Jordan Gaines, Duanping Liao, Edward O. Bixler

**Affiliations:** 1 Sleep Research & Treatment Center, Penn State Milton S. Hershey Medical Center, College of Medicine, Pennsylvania State University, Hershey, PA 17033, USA; scalhoun@psu.edu (S.L.C.); avgontzas@hmc.psu.edu (A.N.V.); yunli@hmc.psu.edu (Y.L.); jgaines1@hmc.psu.edu (J.G.); ebixler@hmc.psu.edu (E.O.B.); 2 Public Health Sciences, College of Medicine, Pennsylvania State University, Hershey, PA 17033, USA; dliao@phs.psu.edu

**Keywords:** adolescents, externalizing behaviors, depression, insomnia, internalizing behaviors, objective sleep duration, phenotypes, rumination

## Abstract

Based on previous studies on the role of objective sleep duration in predicting morbidity in individuals with insomnia, we examined the role of objective sleep duration in differentiating behavioral profiles in adolescents with insomnia symptoms. Adolescents from the Penn State Child Cohort (*n* = 397, ages 12–23, 54.7% male) underwent a nine-hour polysomnography (PSG), clinical history, physical examination and psychometric testing, including the Child or Adult Behavior Checklist and Pediatric Behavior Scale. Insomnia symptoms were defined as a self-report of difficulty falling and/or staying asleep and objective “short” sleep duration as a PSG total sleep time ≤7 h. A significant interaction showed that objective short sleep duration modified the association of insomnia symptoms with internalizing problems. Consistently, adolescents with insomnia symptoms and short sleep duration were characterized by depression, rumination, mood dysregulation and social isolation, while adolescents with insomnia symptoms and normal sleep duration were characterized by rule-breaking and aggressive behaviors and, to a lesser extent, rumination. These findings indicate that objective sleep duration is useful in differentiating behavioral profiles among adolescents with insomnia symptoms. The insomnia with objective short sleep duration phenotype is associated with an increased risk of depression earlier in the lifespan than previously believed.

## 1. Introduction

Sleep problems are highly prevalent during adolescence [1] and negatively affect aspects of physical, emotional, cognitive and social development, impairing both the parent’s and child’s daytime functioning. The most common parent- and self-reported sleep complaint in adolescents is insomnia (difficulty falling and/or staying asleep), with estimates ranging from 11%–47% [2,3,4]. These estimates are particularly alarming given that a great deal of evidence suggests that untreated insomnia may persist into and throughout adulthood [5,6,7].

Furthermore, insomnia symptoms have been shown to be associated with internalizing and externalizing problems in young children [8,9,10,11,12,13], with fewer studies in adolescents [3,5,14]. Emerging evidence suggests that sleep problems in children predict the onset of externalizing problems as well as internalizing problems such as depression and anxiety [8,15,16,17,18]. For example, sleep problems in childhood appear to be a risk factor for anxiety and depression in adulthood [8].

In adults, objective sleep measures are a useful marker in distinguishing two insomnia subtypes; one associated with short sleep duration, activation of stress system and medical morbidity (e.g., hypertension, cognitive impairment), and the other associated with normal sleep duration, lack of stress system activation or medical morbidity [19]. For example, a general population study of adults showed that insomnia with short sleep duration was associated with incident depression independent of poor coping resources, while the association of insomnia with normal sleep duration was not independent of poor coping resources [20]. In addition, a general population study of young children showed that insomnia symptoms with short sleep duration are related to hypothalamic-pituitary-adrenal axis activation when compared to children with insomnia symptoms with “normal” sleep duration or controls [21]. Furthermore, a recent general population study of young children showed that children with insomnia symptoms and short sleep duration were associated with internalizing problems, while children with insomnia symptoms and normal sleep duration were associated with externalizing problems, inattention, hyperactivity, and school problems [9]. However, very limited data is available on the association of insomnia symptoms and short sleep duration with behavioral problems in adolescents [22,23,24]. For example, two very recent studies showed that a sleep duration <7 h was associated with increased externalizing and risky behaviors (e.g., antisocial behavior, drug use, sensation seeking) in early adolescents [24] and that adolescents with insomnia symptoms who slept <7 h had a significantly increased risk of depression [25]. However, there are no studies that have used objective measures of sleep duration and examined in detail the behavioral profiles of adolescents with insomnia symptoms using standardized psychometric testing.

Thus, the aims of this study were threefold: (1) to examine the association of insomnia symptoms with internalizing and externalizing behavior problems; (2) to test the role of objective sleep duration on the association of insomnia symptoms with internalizing and externalizing behavior problems, and (3) to differentiate the behavioral profiles of insomnia phenotypes based on objective sleep duration in adolescents from the general population. We hypothesized that adolescents with insomnia symptoms will have significantly greater internalizing and externalizing behavioral problems and that the association of insomnia symptoms with internalizing problems, particularly depression, will be significantly increased in those with objective short sleep duration.

## 2. Materials and Methods

### 2.1. Participants

The Penn State Child Cohort (PSCC) is a general population sample of 700 children between ages 5–12 years, of whom 421 were followed up 8.4 years later as adolescents (mean age 17.0 ± 2.2 years, 53.9% male, and 21.9% ethnic minority). The loss to follow-up was mainly due to participants moving away from central Pennsylvania; no major differences in the baseline demographic characteristics, however, were observed between those who did and did not participate in the follow-up study [26]. The study protocol was approved by the Penn State University College of Medicine Institutional Review Board. Written informed consents were obtained from participants, as well as their parents or legal guardians if younger than 18 years.

### 2.2. Sleep Laboratory Evaluation

During their laboratory visit, all subjects underwent a series of subjective and objective measurements including parent-reported (subjects’ age <18 years) and self-reported (subjects’ age ≥18 years) questionnaires and rating scales (e.g., behavior, sleep and pubertal development), and measurement of height and weight. Body mass index (BMI) was calculated (in kg/m^2^) and converted to a percentile according to a formula based on the Centers for Disease Control’s sex-specific BMI-for-age growth charts [27]. Participants identified their race/ethnicity from one of six options, as part of the clinical history, and was treated as a dummy variable with white as the reference group vs. ethnic-minority, including Black, Native-American, Asian, Multiple races and Hispanic ethnicity. Parents provided data on their occupation and socioeconomic status (SES) was defined as one or both parents having a professional or managerial occupation vs. both parents having a non-professional or non-managerial occupation, including being unemployed (i.e., low SES).

All participants underwent a single-night, 9-h polysomnography (PSG) recording in a sound-attenuated, light- and temperature-controlled room with a comfortable, bedroom-like atmosphere. Each subject was continuously monitored from 22:00 h until 07:00 h using 14-channel recordings of electroencephalogram (EEG), electrooculogram (EOG), and electromyogram (EMG). Respiration was monitored via nasal pressure (Pro-Tech PTAF Lite; Mukilteo, WA, USA), thermocouple (Salter Labs; Lake Forest, IL, USA), and thoracic/abdominal strain gauges (Model 1312, Sleepmate Technologies; Midlothian, VA, USA). Hemoglobin oxygen saturation (SpO2) was assessed using a pulse oximeter placed on the index finger (Model 3011 Xpod, Nonin Medical, Inc.; Plymouth, MN, USA). Snoring sounds were monitored via a sensor attached to the throat. All data were recorded using Twin Recording & Analysis software (Grass-Telefactor; West Warwick, RI, USA). Visual sleep stage scoring was conducted by a registered PSG technologist according to standardized criteria [28]. Apnea/hypopnea index (AHI), the number of apneas and hypopneas per hour of sleep, and periodic limb movement index (PLMI), the number of periodic limb movements per hour of sleep, were ascertained [26,29]. Sleep parameters derived from PSG included sleep continuity variables such as sleep onset latency (SOL), wake after sleep onset (WASO), total sleep time (TST), and sleep efficiency (SE) as well as sleep architecture parameters defined as the amount of time spent in each sleep stage (i.e., stage 1, stage 2, slow wave sleep [SWS] and rapid eye movement sleep [REM]) divided by total sleep time.

From the PSG-measured TST, we split the overall sample into 3 ordinal groups based on the cohort quartiles: ≥8 h (i.e., 75th percentile), 7–8 h (i.e., median of the sample), and ≤7 h (i.e., 25th percentile). Based on this distribution, which is similar to that reported for this age range in previous studies [30] and consistent with current recommendations for “optimal” sleep as ≥8 h in this age group [31], we defined suboptimal objective sleep duration as PSG-measured TST between 7 and 8 h and objective “short” sleep duration as PSG-measured TST ≤7 h. We should note that these three ordinal groups were mutually exclusive; in other words, all adolescents in the 7–8 h group slept between 7 and 8 h (i.e., >7 h and <8 h).

### 2.3. Insomnia Symptoms and Other Self-Reports

Insomnia symptoms were considered present if participants answered yes to either or both of the following questions: “*do you have difficulty falling asleep?*” (DFA) or “*do you have difficulty staying asleep?*” (DSA) from a self-reported version of the Pediatric Sleep Questionnaire [32]. Pubertal development (Tanner staging) was determined via a self-administered rating scale [33]. Circadian preference was measured using the Morningness-Eveningness Questionnaire (MEQ), which has been validated in both adults and adolescents [34].

### 2.4. Child and Adult Behavior Checklist

Parent- and self-reported questionnaires were administered to measure behaviors including internalizing and externalizing symptoms. All participants older than 18 years competed the self-reported Adult Behavior Checklist (ABCL), while the parents of participants younger than 18 years old completed the Child Behavior Checklist (CBCL) [35,36]. Internalizing and externalizing scales from the C/ABCL were used to measure the severity of anxious depressed (e.g., “*nervous*”, “*worries*”, “*cries*”, “*worthless*”), withdrawn depressed (e.g., “*sad*”, “*withdrawn*”, “*enjoys little*”, “*prefers to be alone*”), and somatic complaints (e.g., “*headaches*”, “*stomach ache*”, “*nausea*”, “*tired*”) as well as thought problems (e.g., “*can’t get mind off of certain thoughts*”, “*strange ideas*”, “*strange behavior*”), attention problems (e.g., “*poor concentration*”, “*fails to finish tasks*”, “*daydreams*”, “*impulsivity*”), rule-breaking behaviors (e.g., “*breaks rules*”, “*lies and cheats*”, “*lack of guilt*”, “*uses drugs*”), and aggressive behaviors (e.g., “*fights*”, “*screams a lot*”, “*mood changes*”, “*loses temper*”). For each scale and subscale, T-scores with a mean of 50 and a standard deviation of 10 are obtained following standard procedures [35,36]. A T-score ≥60 is considered a clinically meaningful elevation for the C/ABCL scales and subscales in general population samples [35,36]. Clinically elevated internalizing and externalizing scores were used as primary outcomes in the present study, while each subscale T-scores were used in behavioral profiles.

### 2.5. Pediatric Behavior Scale

The parents of all participants completed the Pediatric Behavior Scale (PBS) [37]—a 165-item rating scale that assesses behaviors over the past 2 months, including Aggression (e.g., “*mean or cruel to others*”, “*threatens, bullies*”, “*starts fights*”, “*lies, cheats or steals*”), Inappropriate Social Behavior (e.g., “*immature*”, “*pesters or nags*”, “*loud*”, “*poor social judgment*”), Perseverative Thinking (e.g., “*hard to get an idea out of his/her mind*”, “*talks about the same things over and over*”, “*upset by changes in routine*”), Social Isolation (e.g., “*doesn’t get along with other children*”, “*hard time making friends*”, “*withdrawn*, *spends a lot of time alone*”, “*doesn’t take part in normal social activities*”), and Thought Disorder (e.g., “*sees or hears things that aren’t really there*”, “*says strange things that don’t make sense*”, “*strange, unusual or bizarre behavior*”, “*very suspicious of others*”). For each subscale, a total raw score was obtained following standard procedures. The PBS has been used in several studies in children from the general population and with a wide range of behavioral disorders [9,12,13,21,38] and it served in the present study as an external validator of specific behavioral problems included in the Thought Problems and Aggressive Behaviors subscales of the C/ABCL.

### 2.6. Statistical Analyses

The sample was divided into two groups based on presence or absence of insomnia symptoms. Comparisons of the distribution of demographics according to group membership were made with *x*^2^ tests or analysis of variance. A two-way multivariate analysis of covariance (MANCOVA) was used to examine the association of insomnia symptoms (no vs. yes), objective sleep duration (i.e., ≥8, 7–8, and ≤7 h of sleep), and their interaction on our primary outcomes of clinically elevated internalizing and externalizing problems while controlling for age, race, gender, SES, BMI percentile, eveningness, AHI, and PLMI. The association of insomnia symptoms with internalizing and externalizing problems was plotted based on the objective sleep duration strata (i.e., ≥8, 7–8, and ≤7 h of sleep). Based on these finding, all adolescents were separated in post-hoc analyses based on their self-reported insomnia symptoms status (no vs. yes) and objective “short” (i.e., ≤7 h) vs. “normal” (i.e., >7 h) sleep duration, yielding four groups: controls with normal sleep duration (*n* = 188), controls with short sleep duration (*n* = 61), insomnia symptoms with normal sleep duration (*n* = 113), and insomnia symptoms with short sleep duration (*n* = 35). Given the lack of differences among controls with normal and short sleep duration, these subgroups were merged back together. To study the behavioral profiles across subgroups, we examined the C/ABCL subscales scores across controls and insomnia symptoms subgroups based on objective sleep duration. Finally, we conducted item-level (C/ABCL) as well as external validity (PBS) analyses for specific behavioral problems. The critical statistical confidence level for all analyses was *p* < 0.05 and two-tailed. Analyses were performed using IBM SPSS Statistics for Windows, Version 23 (IBM Corp., Armonk, NY, USA).

## 3. Results

### 3.1. Demographic and Clinical Characteristics of the Sample

Out of the 421 adolescents, we excluded from the analyses 17 adolescents who replied “do not know” to both insomnia symptoms questions and 7 adolescents who did not complete the parent- or self-reported C/ABCL; thus, 397 adolescents were included in the final analyses. Sociodemographic characteristics by insomnia symptoms and objective sleep duration groups are presented in Table 1. Insomnia symptoms were significantly associated with female gender, low SES, and eveningness, while objective short sleep duration was significantly associated with male gender but no other significant sociodemographic differences were found. Furthermore, insomnia symptoms were not significantly associated with sleep continuity or architecture parameters, except slightly longer sleep onset latency and lower periodic limb movements (see Table 1). As expected, objective sleep duration groups were associated with significant differences in all PSG parameters, except amount of stage 2, stage 3, periodic limb movements and apneas or hypopneas.

### 3.2. Internalizing and Externalizing Problems

**Figure 1:**
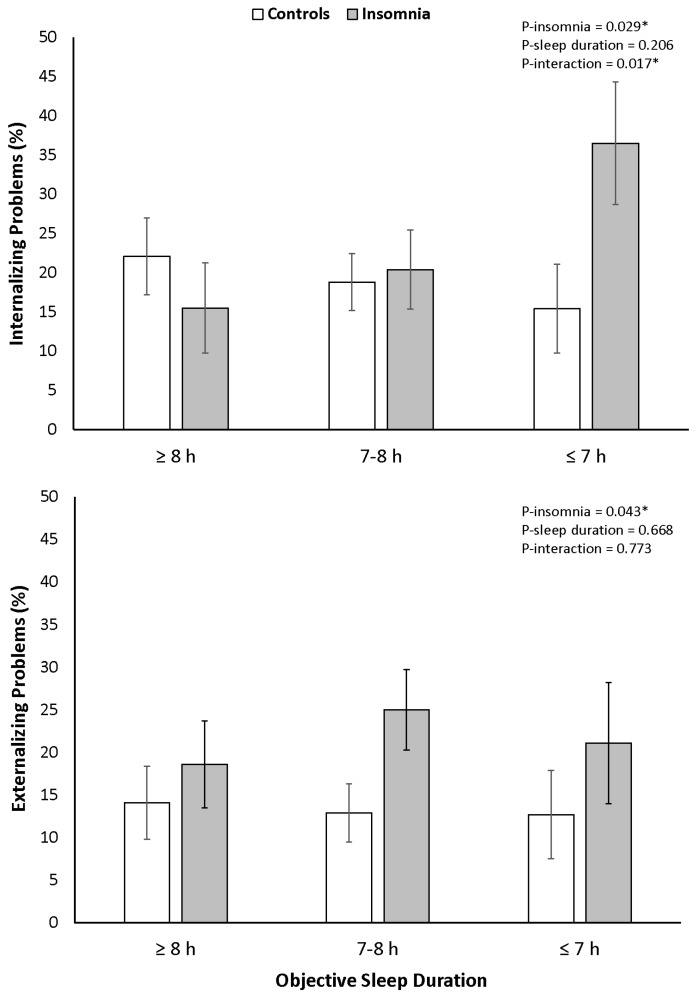
Clinically elevated internalizing and externalizing problems associated with insomnia symptoms across objective sleep duration groups. A significant interaction revealed that the association of insomnia symptoms with internalizing problems was primarily present in those with objective “short” sleep duration (i.e., ≤7 h), while insomnia symptoms among those with “normal” sleep duration (i.e., >7 h) was not significantly associated with internalizing problems. There was no significant interaction on externalizing problems but a significant main effect of insomnia symptoms. All data adjusted for sex, age, race, socioeconomic status, body mass index percentile, morning-eveningness questionnaire score, apnea/hypopnea index and periodic limb movement index.

As shown in Table 1, adolescents with insomnia symptoms showed more clinically elevated internalizing (F_1,395_ = 4.77, *p* = 0.029, _p_η^2^ = 0.012) and externalizing (F_1,395_ = 4.14, *p* = 0.043, _p_η^2^ = 0.011) problems as compared to controls, while no differences across objective sleep duration groups were observed (F_2,395_ = 1.60, *p* = 0.206, _p_η^2^ = 0.004 and F_2,395_ = 0.40, *p* = 0.668, _p_η^2^ = 0.002, respectively). A significant interaction revealed that objective sleep duration modified the association between insomnia symptoms and internalizing problems (F_2,395_ = 4.10, *p* = 0.017, _p_η^2^ = 0.018), even after controlling for age, gender, race, SES, BMI percentile, eveningness, AHI, and PLMI. The interaction on clinically elevated externalizing problems was not statistically significant (F_2,395_ = 0.26, *p* = 0.773, _p_η^2^ = 0.001). As shown in Figure 1, insomnia symptoms were significantly associated with clinically elevated internalizing problems among those who slept objectively ≤7 h (36.5% vs. 15.4%, *p* = 0.046), while insomnia symptoms were not significantly associated with clinically elevated internalizing problems among those who slept objectively ≥8 h (15.5% vs. 22.1%, *p* = 0.398) or 7–8 h (20.4% vs. 18.8%, *p* = 0.800). Based on these data, we stratified adolescent controls and with insomnia symptoms into those with “short” (i.e., ≤7 h) and “normal” (i.e., >7 h) objective sleep duration.

### 3.3. Behavioral Profiles

**Figure 2:**
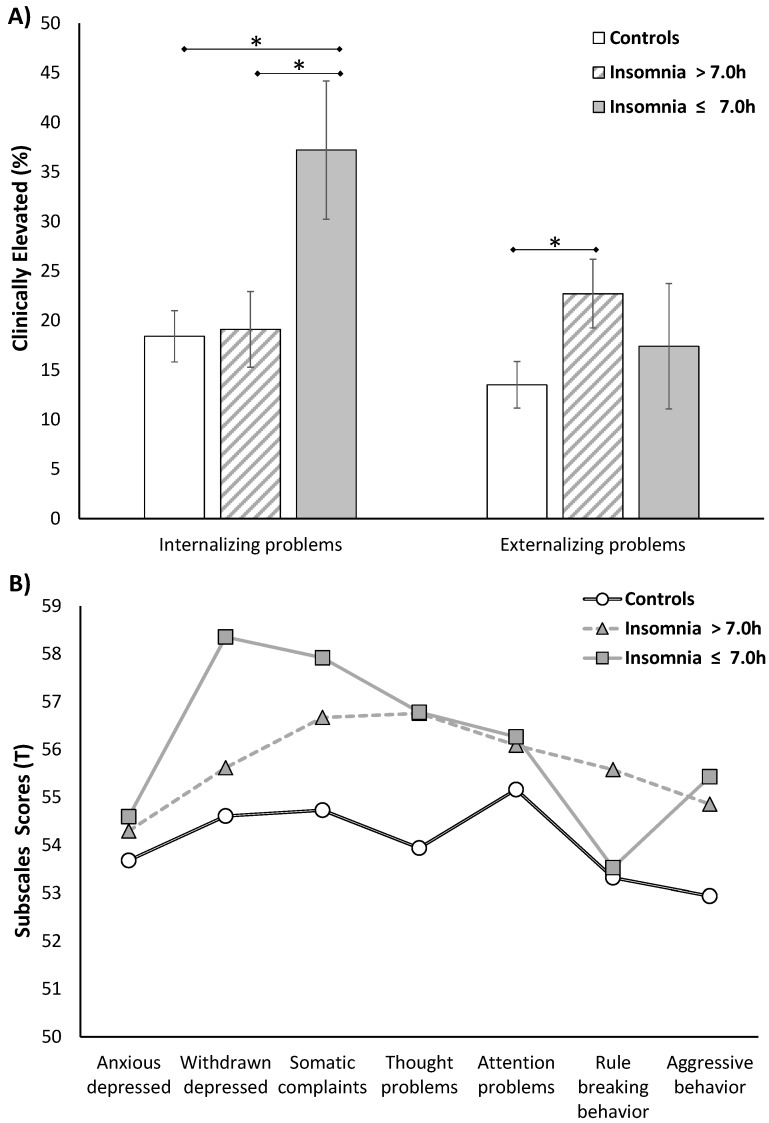
Behavioral profiles of insomnia symptoms phenotypes based on objective sleep duration. (**A**) Adolescents with insomnia symptoms who slept objectively ≤7 h showed clinically elevated internalizing problems as compared to controls; in contrast, adolescents with insomnia symptoms who slept objectively >7 h showed clinically elevated externalizing problems as compared to controls; (**B**) The behavioral profile of internalizing and externalizing subscales was significantly different between insomnia phenotypes when compared to controls. Adolescents with insomnia symptoms who slept objectively ≤7 h had significantly higher withdrawn depression, somatic complaints, thought problems (rumination) and aggressive behaviors (mood regulation) as compared to controls, while adolescents with insomnia symptoms who slept objectively >7 h had significantly higher somatic complaints, thought problems (rumination), rule-breaking and aggressive behaviors (acting-out aggression) as compared to controls. All data adjusted for sex, age, race, socioeconomic status, body mass index percentile, morning-eveningness questionnaire score, apnea/hypopnea index and periodic limb movement index.

We examined the behavioral profiles based on the C/ABCL subscales comprising internalizing and externalizing problems across insomnia symptoms subgroups, while adjusting for age, gender, race, SES, BMI percentile, eveningness, AHI, and PLMI, and significant differences were found on withdrawn depression (F_3,395_ = 2.85, *p* = 0.037, _p_η^2^ = 0.022), somatic complaints (F_3,395_ = 3.56, *p* = 0.014, _p_η^2^ = 0.027), thought problems (F_3,395_ = 6.12, *p* = 0.0005, _p_η^2^ = 0.046), rule-breaking behaviors (F_3,395_ = 4.56, *p* = 0.004, _p_η^2^ = 0.035) and aggressive behaviors (F_3,395_ = 4.48, *p* = 0.004, _p_η^2^ = 0.034). As shown in Table 2, adolescents with insomnia symptoms who slept objectively ≤7 h had significantly higher scores on withdrawn depression, somatic complaints, thought problems, and aggressive behaviors as compared to controls with normal sleep duration, while adolescents with insomnia symptoms who slept objectively >7 h had significantly higher scores on somatic complaints, thought problems, rule-breaking behaviors, and aggressive behaviors as compared to controls with normal sleep duration. Importantly, withdrawn depression was a key feature of adolescents with insomnia symptoms who slept objectively ≤7 h, while rule-breaking behaviors were a key feature of adolescents with insomnia symptoms who slept objectively >7 h when compared to each other (*p* = 0.049 and *p* = 0.051, respectively). As shown in Figure 2A, adolescents with insomnia symptoms who slept objectively ≤7 h had clinically elevated internalizing problems, while adolescents with insomnia symptoms who slept objectively >7 h had clinically elevated externalizing problems as compared to controls. As shown in Figure 2B, the behavioral profiles based on each of the internalizing and externalizing subscales were significantly different across insomnia symptoms subgroups based on objective sleep duration. Controls, regardless of objective sleep duration, were used as the reference group in Figure 2 given the lack of association of objective sleep duration with any behavioral scale or subscale within this group (Table 2).

### 3.4. Rumination, Internalization and Acting-Out

As mentioned above, adolescents with insomnia symptoms with either normal or short sleep duration were significantly associated with thought problems and aggressive behaviors as compared to controls (see Table 2 and Figure 2B). However, these C/ABCL subscales include diverse items such as “*can’t get mind off of certain thoughts*”, “*strange ideas”* and “*strange behavior*” among thought problems and “*fights*”, “*screams a lot*”, “*mood changes*” and “*loses temper*” among aggressive behaviors. To better understand these associations, we examined the relative contribution of specific items to these scores. As shown in Table 3, we found that both insomnia symptoms subgroups significantly endorsed the item “*can’t get mind off of certain thoughts*” of the thought problems subscale (*p* = 0.009 and *p* = 0.017, respectively) but did not significantly endorsed the items “*strange ideas*” (*p* = 0.181 and *p* = 0.115, respectively) or “*strange behavior*” (*p* = 0.095 and *p* = 0.216, respectively) as compared to controls. In contrast, adolescents with insomnia symptoms who slept objectively >7 h significantly endorsed the items “*fights*” and “*screams a lot*” of the aggressive behaviors subscale (*p* = 0.009 and *p* = 0.011, respectively), while adolescents with insomnia symptoms who slept objectively ≤7 h did not (*p* = 0.846 and *p* = 0.309, respectively). In fact, this latter insomnia symptoms subgroup significantly endorsed the items “*mood changes*” and “*loses temper*” of the aggressive behaviors subscale (*p* = 0.014 and *p* = 0.037, respectively), which indicated that the elevated aggressive behaviors in adolescents with insomnia symptoms who slept objectively ≤7 h were driven by mood-related items consistent with their elevated withdrawn depression, rather than acting-out aggression.

Finally, we further validated our findings using an external measure, the PBS, which provides a detailed assessment of aggression, inappropriate social behavior, perseverative thinking, social isolation and thought disorder by parent-report (see Table 3). Commensurate with our C/ABCL subscales and item-level findings, we found that adolescents with insomnia symptoms who slept objectively >7 h were significantly associated with elevated scores in aggression and inappropriate social behavior as compared to controls (*p* = 0.011 and *p* = 0.001, respectively), while adolescents with insomnia symptoms who slept objectively ≤7 h were not (*p* = 0.871 and *p* = 0.242, respectively). In contrast, adolescents with insomnia symptoms who slept objectively ≤7 h were significantly associated with elevated scores in perseverative thinking and social isolation as compared to controls (*p* = 0.019 and *p* = 0.001, respectively), while adolescents with insomnia symptoms who slept objectively >7 h were marginally or not significantly associated with these behavioral problems (*p* = 0.062 and *p* = 0.288, respectively). Neither adolescent insomnia symptoms subgroup with either normal or short sleep duration showed significantly elevated thought disorder scores as compared to controls on the PBS (*p* = 0.119 and *p* = 0.801, respectively).

## 4. Discussion

This general population study demonstrated that adolescents with insomnia symptoms and objective short sleep duration had a behavioral profile characterized by withdrawn depression and rumination, whereas adolescents with insomnia symptoms and normal sleep duration had a behavioral profile with prominent externalizing behaviors such as rule-breaking and aggressive behaviors. This is the first study to examine the moderating role of objectively-measured sleep duration on the behavioral profiles of adolescents with insomnia symptoms. These findings further support the validity of insomnia phenotypes based on objective sleep duration as early as adolescence.

Previous studies have shown that adolescents with insomnia symptoms report more behavioral and emotional problems than good sleepers [14]. The novelty of the present study resides in the use of objective sleep duration to define insomnia phenotypes in adolescents. Despite the fact that we have proposed that the insomnia with objective short sleep duration phenotype in adults may carry greater biological vulnerability and an early onset in life [19], very limited data is available on this insomnia phenotype in children and adolescents. The findings of the present study are consistent with our previous studies in this cohort when participants were pre-pubertal children 5 to 12 years old. We previously showed that these young children with parent-reported insomnia symptoms and objective short sleep duration had increased evening and morning cortisol levels [21] and a behavioral profile characterized by internalizing behaviors (e.g., anxiety and depression) [9], while children with parent-reported insomnia symptoms and normal sleep duration had normal evening and morning cortisol levels [21] and a behavioral profile consistent with limit-setting and rule-breaking behaviors [9]. The same behavioral pattern was observed in adolescents with insomnia symptoms based on objective sleep duration. Some difference in the behavioral profiles between childhood insomnia and adolescence insomnia are expected, given that, for example, inattention/hyperactivity problems may be more strongly associated with insomnia symptoms in childhood [9] and adolescents can express more clearly depressive symptoms such as sadness, hopelessness, helplessness, social withdrawal, etc. The present findings are also consistent with two recent studies that found that adolescents with insomnia symptoms who slept less than six hours had an eight-fold increased odds of depression [25] and that insomnia with objective short sleep duration in adults is associated with incident depression independent of poor coping resources, whereas the association of insomnia with normal sleep duration with incident depression was explained by their poor coping skills [20]. Together, these findings suggest that adolescence may be a critical developmental period for the association of insomnia with depression, particularly among those with objective short sleep duration.

In the present study, we were able to demonstrate that the clinically elevated aggressive behaviors and thought problems captured by the C/ABCL were in fact related to overt rule-breaking and aggressive behaviors in the insomnia symptoms with normal sleep duration phenotype, while these elevations were related to rumination and emotion regulation (rather than psychotic-like thought problems) in the insomnia symptoms with short sleep duration phenotype. Indeed, an interesting finding of the present study was that rumination, as measured by C/ABCL thought problems’ items and PBS perseverative thinking subscale, was associated with both insomnia symptoms phenotypes, albeit to a differing degree, in these adolescents. It appears that rumination is a prominent characteristic of all adolescents and adults with insomnia [39,40,41] and may reflect cortical hyperarousal, as measured by high-frequency EEG dynamics in the beta range (15–35 Hz) during sleep, which is present in both insomnia phenotypes [42]. We hypothesize that disturbed cortical networks, regardless of objective sleep duration, may be a shared mechanism putting adolescents with insomnia symptoms at risk of diverse psychiatric disorders.

Our findings suggest that insomnia symptoms with objective short duration in adolescents is associated with cognitive-emotional hyperarousal, dysfunctional emotion regulation (i.e., anger-in) [43], greater risk of mood disorders such as clinical depression, and is in a continuum with adult insomnia associated with physiologic hyperarousal [19], whereas adolescents with insomnia symptoms and normal sleep duration are more likely to be associated with poor lifestyle and sleep-incompatible habits driven by their rule-breaking and acting-out behaviors. Furthermore, it appears that objective sleep measures can be useful in phenotyping insomnia across the lifespan, in contrast to the recent shift towards simplifying insomnia nosology, in that insomnia disorder is no longer separated into discrete diagnostic categories. The proposed phenotyping of insomnia based on neuroendocrine (e.g., cortisol) and neurophysiologic data (e.g., high-frequency EEG) and behavioral profiles may lead to better diagnostic reliability and validity and development of phenotype-based treatments across the lifespan.

Several limitations should be considered when interpreting the results of our study. First, the study is cross-sectional and does not allow causal conclusions. It is likely that the relationship between sleep disturbance and behavior problems is complex and bidirectional. Second, the objective sleep duration in this study was based on one night of fixed-time PSG, which may not be representative of the subjects’ habitual sleep duration and may be affected by first-night effect. Moreover, there is the possibility that adolescents with internalizing disorders may be more susceptible to the in-lab PSG conditions without an adaptation night. However, recent studies have shown that one night of PSG is useful in the relative classification of insomnia subjects as short or normal sleepers [44]. Our cut-offs for objective sleep duration were based on an empirical approach (i.e., quartiles of sleep duration of the sample), which provide a relative classification among adolescents without assuming a priori cut-offs and are consistent with current definitions of optimal and inadequate hours of sleep [24,31]. Future studies should explore the optimal cut-off of sleep duration based on clinical criteria and explore the use of multiple PSG nights or less expensive objective measures of habitual sleep (e.g., actigraphy). Third, the definition of insomnia was that of symptoms rather than a disorder or syndrome, as it did not include general diagnostic criteria (ICSD-3), nor did we exclude other highly prevalent sleep disorders in this age group (e.g., delayed sleep phase disorder, sleep disordered breathing). Nevertheless, we controlled for the potential effect of evening circadian preference (MEQ), sleep disordered breathing (AHI) and periodic limb movements (PLMI). Future large, epidemiological studies should use current diagnostic criteria for insomnia disorder, as with such definitions, differences among subgroups may be even more pronounced, particularly in terms of effect size. Despite these limitations, this study extends the limited previous knowledge on insomnia in adolescents using a random general population sample and objective sleep measures.

## 5. Conclusions

In summary, adolescents with insomnia symptoms have clinically elevated externalizing and internalizing behavioral problems. Importantly, adolescents with insomnia symptoms and objective normal sleep duration have more externalizing behaviors such as rule-breaking and aggressive behaviors, while adolescents with insomnia symptoms and objective short sleep duration have clinically elevated internalizing problems, particularly withdrawn depression and rumination. These differential behavioral profiles, together with accumulating physiologic data, suggest that phenotyping of insomnia could be introduced as early as adolescence and childhood. An improved insomnia nosology based on objective sleep measures may provide clinicians with the ability to differentiate phenotypes and their associated behaviors without relying first-hand on expensive, time-consuming sleep studies. This phenotyping will better guide clinical evaluation and application of specific insomnia interventions such as cognitive behavioral treatment (CBT-I) or hypnotic medication.

## Figures and Tables

**Table 1:** Demographic, clinical and polysomnographic characteristics of the sample stratified by insomnia symptoms and objective sleep duration.

	Insomnia Symptoms		*p*	Objective Sleep Duration			*p*
	No	Yes		≥8 h	7–8 h	≤7 h	
	(*n* = 249)	(*n* = 148)		(*n* = 120)	(*n* = 181)	(*n* = 96)	
Female, %	37.8	58.1	<0.001	54.2	43.6	37.5	0.042
Ethnic-Minority, %	19.3	24.3	0.234	20.8	22.7	18.8	0.747
Low SES, %	60.9	73.6	0.010	69.7	61.3	68.8	0.247
Age, years	16.9 ± 2.3	17.1 ± 2.2	0.368	16.7 ± 2.2	17.1 ± 2.4	17.1 ± 2.1	0.190
Tanner, stage	4.1 ± 0.8	4.25 ± 0.81	0.195	4.2 ± 0.8	4.2 ± 0.8	4.1 ± 0.8	0.883
BMI percentile	64.4 ± 28.6	68.16 ± 27.9	0.198	65.9 ± 29.4	66.6 ± 26.0	64.1 ± 31.3	0.788
MEQ, total score	26.7 ± 4.7	24.4 ± 5.3	<0.001	25.9 ± 4.7	26.2 ± 5.2	25.2 ± 5.3	0.246
M-type	37.9	22.3	<0.001	28.3	36.5	28.4	0.514
I-type	37.9	35.1		39.2	35.4	36.8	
E-type	24.2	42.6		32.5	28.2	34.7	
SOL, minutes	23.7 ± 19.1	28.6 ± 30.4	0.080	12.7 ± 6.5	23.9 ± 14.2	44.5 ± 37.3	<0.001
Awakenings, number	36.5 ± 11.3	36.7 ± 13.6	0.565	32.9 ± 8.6	39.0 ± 12.1	36.4 ± 14.9	<0.001
WASO, minutes	71.4 ± 44.9	66.0 ± 41.5	0.238	33.0 ± 10.0	63.9 ± 19.6	125.2 ± 47.2	<0.001
TWT, minutes	92.9 ± 51.8	92.8 ± 59.1	0.982	44.1 ± 11.3	86.0 ± 18.3	166.9 ± 54.6	<0.001
TST, minutes	447.2 ± 52.4	448.2 ± 60.0	0.866	497.1 ± 10.5	454.7 ± 18.1	372.2 ± 55.4	<0.001
Sleep efficiency, %	82.8 ± 9.6	82.3 ± 11.0	0.961	91.8 ± 2.1	84.1 ± 3.4	68.9 ± 10.2	<0.001
Stage 1, %	1.0 ± 1.3	1.0 ± 1.9	0.712	0.49 ± 0.5	0.9 ± 1.0	1.9 ± 2.5	<0.001
Stage 2, %	53.5 ± 9.8	53.4 ± 10.3	0.736	52.72 ± 9.5	53.7 ± 9.7	54.0 ± 10.9	0.614
Stage 3, %	26.9 ± 9.4	27.0 ± 8.1	0.936	27.0 ± 8.7	26.4 ± 9.2	28.0 ± 9.6	0.399
Stage R, %	18.6 ± 4.7	18.6 ± 5.6	0.913	19.8 ± 4.4	19.1 ± 4.5	16.1 ± 6.0	<0.001
PLMI, events/hour	4.4 ± 6.5	3.0 ± 4.8	0.014	3.4 ± 5.7	3.8 ± 5.8	4.7 ± 6.6	0.299
AHI, events/hour	2.4 ± 3.1	2.7 ± 4.3	0.379	2.1 ± 3.5	2.5 ± 2.7	2.9 ± 5.0	0.271
Internalizing problems							
T-score, total	49.6 ± 10.1	52.8 ± 11.1	0.004	51.1 ± 9.5	50.5 ± 10.9	50.9 ± 11.4	0.907
Clinically elevated, %	17.3	25.0	0.064	19.2	19.3	22.9	0.741
Externalizing problems							
T-score, total	47.5 ± 9.3	51.5 ± 11.0	<0.001	49.1 ± 9.8	49.0 ± 10.1	48.9 ± 10.7	0.990
Clinically elevated, %	12.0	23.6	0.002	15.8	17.1	15.6	0.933

Data are mean ± standard deviation, unless otherwise stated. AHI = apnea hypopnea index. BMI% = body mass index percentile. E-type = evening chronotype. I-type = intermediate chronotype. MEQ = morningness eveningness questionnaire. M-type = morning chronotype. SES = socioeconomic status. SOL = sleep onset latency. PLMI = periodic leg movements index. Stage R = rapid eye movement sleep. TST = total sleep time. TWT = total wake time. WASO = wake after sleep onset.

**Table 2:** Behavioral profiles of the sample stratified by insomnia symptoms and objective sleep duration subgroups.

	1. Controls >7 h	2. Controls ≤7 h	3. Insomnia >7 h	4. Insomnia ≤7 h	*p*			
	(*n* = 188)	(*n* = 61)	(*n* = 113)	(*n* = 35)	1 vs. 2	1 vs. 3	1 vs. 4	3 vs. 4
Internalizing problems								
Anxious depressed								
T-score, total	53.7 ± 0.4	53.8 ± 0.8	54.3 ± 0.6	54.6 ± 1.0	0.892	0.369	0.401	0.796
Clinically elevated, %	14.9	17.2	20.7	18.4	0.675	0.207	0.621	0.759
Withdrawn depressed								
T-score, total	54.8 ± 0.5	54.1 ± 0.9	55.6 ± 0.8	58.4 ± 1.2	0.553	0.318	0.008	0.049
Clinically elevated, %	20.4	20.3	24.3	37.7	0.990	0.445	0.034	0.110
Somatic complaints								
T-score, total	54.9 ± 0.5	54.1 ± 0.9	56.7 ± 0.6	57.9 ± 1.2	0.392	0.035	0.020	0.342
Clinically elevated, %	18.4	19.3	24.1	30.5	0.881	0.251	0.122	0.427
Externalizing problems								
Thought problems								
T-score, total	54.1 ± 0.5	53.6 ± 1.0	56.8 ± 0.6	56.8 ± 1.1	0.607	<0.01	0.019	0.981
Clinically elevated, %	13.9	15.3	30.5	29.8	0.809	<0.01	0.032	0.934
Attention problems								
T-score, total	55.2 ± 0.5	55.1 ± 0.9	56.1 ± 0.7	56.3 ± 1.2	0.975	0.281	0.410	0.896
Clinically elevated, %	18.8	16.0	23.3	25.6	0.650	0.353	0.367	0.764
Rule-breaking behaviors								
T-score, total	53.4 ± 0.9	53.1 ± 0.7	55.6 ± 0.5	53.5 ± 0.3	0.727	<0.01	0.883	0.051
Clinically elevated, %	12.8	10.0	23.2	19.7	0.598	0.018	0.314	0.621
Aggressive behaviors								
T-score, total	52.7 ± 0.4	53.6 ± 0.7	54.9 ± 0.5	55.4 ± 1.0	0.195	<0.01	0.010	0.604
Clinically elevated, %	9.5	17.5	19.1	26.1	0.123	0.024	0.013	0.302

Data are means ± standard errors of total T-scores and clinically elevated T-scores adjusted for sex, age, race, socioeconomic status, body mass index percentile, morning-eveningness questionnaire score, apnea/hypopnea index and periodic limb movement index.

**Table 3:** Item-level analysis of the Child / Adult Behavior Checklist (C/ABCL) and related subscales of the Pediatric Behavior Scale (PBS) across insomnia symptoms and objective sleep duration subgroups.

	1. Controls	2. Insomnia >7 h	3. Insomnia ≤7 h	*p*		
				1 vs. 2	1 vs. 3	2 vs. 3
**C/ABCL**	(*n* = 247)	(*n* = 113)	(*n* = 35)			
Aggressive behavior						
“fights”	4.8%	12.4%	5.7%	0.009	0.846	0.176
“screams a lot”	8.4%	17.7%	14.3%	0.011	0.309	0.580
“mood changes”	25.3%	38.9%	45.7%	0.009	0.014	0.445
“loses temper”	20.9%	30.1%	37.1%	0.060	0.037	0.398
Thought problems						
“can’t get mind off of certain thoughts”	33.3%	47.8%	54.3%	0.009	0.017	0.488
“strange ideas”	6.4%	10.6%	14.3%	0.181	0.115	0.492
“strange behavior”	7.6%	13.3%	14.3%	0.095	0.216	0.861
**PBS**	(*n* = 217)	(*n* = 93)	(*n* = 29)			
Aggression	0.81 ± 0.16	1.54 ± 0.24	0.88 ± 0.43	0.011	0.871	0.177
Inappropriate Behavior	1.82 ± 0.26	3.36 ± 0.39	2.73 ± 0.72	0.001	0.242	0.432
Social Isolation	0.71 ± 0.13	0.93 ± 0.19	1.97 ± 0.35	0.288	0.001	0.011
Perseverative Thinking	0.66 ± 0.11	1.04 ± 0.17	1.43 ± 0.31	0.062	0.019	0.256
Thought Disorder	0.09 ± 0.04	0.20 ± 0.06	0.07 ± 0.10	0.119	0.801	0.246

Child/Adult Behavior Checklist data are percent of subjects who endorsed that item. Pediatric Behavior Scale data are mean raw scores ± standard errors adjusted for sex, age, race, socioeconomic status, body mass index percentile, morning-eveningness questionnaire score, apnea/hypopnea index and periodic limb movement index.
